# Correlation of Ventricular Arrhythmogenesis with Neuronal Remodeling of Cardiac Postganglionic Parasympathetic Neurons in the Late Stage of Heart Failure after Myocardial Infarction

**DOI:** 10.3389/fnins.2017.00252

**Published:** 2017-05-08

**Authors:** Dongze Zhang, Huiyin Tu, Chaojun Wang, Liang Cao, Robert L. Muelleman, Michael C. Wadman, Yu-Long Li

**Affiliations:** ^1^Department of Emergency Medicine, University of Nebraska Medical CenterOmaha, NE, USA; ^2^Department of Cardiovascular Disease, The First Affiliated Hospital of Xi'an Jiaotong UniversityXi'an, China; ^3^Department of Cardiac Surgery, Second Xiangya Hospital, Central South UniversityChangsha, China; ^4^Department of Cellular & Integrative Physiology, University of Nebraska Medical CenterOmaha, NE, USA

**Keywords:** chronic heart failure, electrocardiogram, intracardiac ganglia, N-type calcium currents, parasympathetic, ventricular arrhythmia

## Abstract

**Introduction:** Ventricular arrhythmia is a major cause of sudden cardiac death in patients with chronic heart failure (CHF). Our recent study demonstrates that N-type Ca^2+^ currents in intracardiac ganglionic neurons are reduced in the late stage of CHF rats. Rat intracardiac ganglia are divided into the atrioventricular ganglion (AVG) and sinoatrial ganglion. Only AVG nerve terminals innervate the ventricular myocardium. In this study, we tested the correlation of electrical remodeling in AVG neurons with ventricular arrhythmogenesis in CHF rats.

**Methods and Results:** CHF was induced in male Sprague-Dawley rats by surgical ligation of the left coronary artery. The data from 24-h continuous radiotelemetry ECG recording in conscious rats showed that ventricular tachycardia/fibrillation (VT/VF) occurred in 3 and 14-week CHF rats but not 8-week CHF rats. Additionally, as an index for vagal control of ventricular function, changes of left ventricular systolic pressure (LVSP) and the maximum rate of left ventricular pressure rise (LV dP/dt_max_) in response to vagal efferent nerve stimulation were blunted in 14-week CHF rats but not 3 or 8-week CHF rats. Results from whole-cell patch clamp recording demonstrated that N-type Ca^2+^ currents in AVG neurons began to decrease in 8-week CHF rats, and that there was also a significant decrease in 14-week CHF rats. Correlation analysis revealed that N-type Ca^2+^ currents in AVG neurons negatively correlated with the cumulative duration of VT/VF in 14-week CHF rats, whereas there was no correlation between N-type Ca^2+^ currents in AVG neurons and the cumulative duration of VT/VF in 3-week CHF.

**Conclusion:** Malignant ventricular arrhythmias mainly occur in the early and late stages of CHF. Electrical remodeling of AVG neurons highly correlates with the occurrence of ventricular arrhythmias in the late stage of CHF.

## Introduction

Ventricular arrhythmia, including ventricular tachycardia and ventricular fibrillation (VT/VF), is the most common cause of sudden cardiac death (SCD) in patients with chronic heart failure (CHF) (Tomaselli and Zipes, 2004; Kolettis et al., 2015). Apart from structural and electrophysiological remodeling of the ventricle (Tomaselli and Zipes, 2004; Yamada et al., 2016), neuronal remodeling in the autonomic nervous system also plays a pivotal role in the development and maintenance of ventricular arrhythmias in patients with CHF and in CHF animal models (Ogawa et al., 2007; Vaseghi and Shivkumar, 2008; Shen and Zipes, 2014; Ajijola et al., 2015). It is well known that autonomic nervous dysfunction is associated with the development and progression of CHF (Fukuda et al., 2015). Much evidence has shown that sympathetic overactivation and withdrawal of parasympathetic activity serve as negative prognostic indicators for high mortality in CHF (Nolan et al., 1998; Frenneaux, 2004; Cygankiewicz et al., 2008; Hauptman et al., 2012; Shen and Zipes, 2014). Our recent study demonstrates that N-type Ca^2+^ currents and cell excitability of intracardiac ganglionic neurons are reduced in the late stage of CHF rats (Tu et al., 2014). Acetylcholine released from intracardiac ganglionic nerve terminals can bind to muscarinic acetylcholine receptors to regulate cardiac function (Thomas, 2011), and Ca^2+^ influx through voltage-gated Ca^2+^ channels is a key trigger for acetylcholine release (Akiyama and Yamazaki, 2000). Rat intracardiac ganglia are divided into the atrioventricular ganglion (AVG) and sinoatrial ganglion. The ventricular myocardium only receives projection of nerve terminals from the AVG (Pardini et al., 1987). These facts indicate that neuronal remodeling of the AVG is possibly involved in ventricular arrhythmogenesis in the CHF state.

Myocardial infarction (MI) is a major cause of CHF in the general population (Wolk et al., 1972; Spencer et al., 1999; Jhund and McMurray, 2008). However, progressive alterations of arrhythmic substrates and potential mechanisms involved in ventricular arrhythmogenesis in post-MI are still unknown. Using a rat model of CHF induced by surgical ligation of the left coronary artery, in this study we investigated the occurrence of ventricular arrhythmias and related arrhythmic substrates during the progression of CHF. We also tested whether electrical remodeling of AVG neurons is related to ventricular arrhythmogenesis during development of CHF.

## Materials and methods

All experimental procedures were approved by the University of Nebraska Medical Center Institutional Animal Care and Use Committee and were carried out in accordance with the National Institutes of Health (NIH Publication No. 85-23, revised 1996) and the American Physiological Society's “Guides for the Care and Use of Laboratory Animals.”

### Animal model

**Figure 1:**
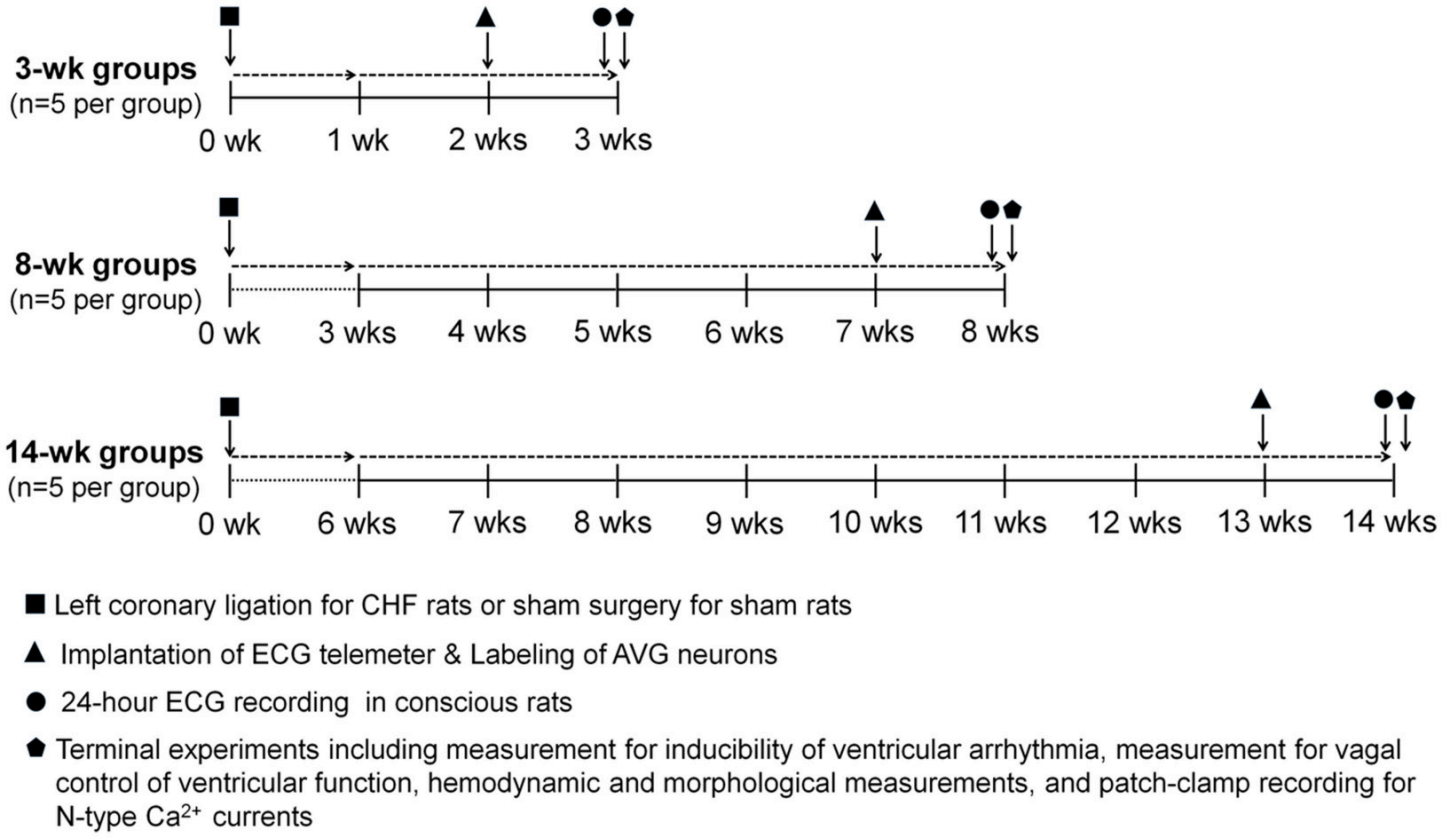
**Schematic diagram illustrating study design, timeline and interventions**. Chronic heart failure (CHF) model was induced by surgical ligation of the left coronary artery in rats. Sham rats underwent the same surgery without left coronary ligation. Implantation of the ECG telemeter and labeling of atrioventricular ganglion (AVG) neurons were performed on the day following 2, 7, or 13 weeks after left coronary ligation (CHF groups) or same surgery (Sham groups). One week after implantation of the ECG telemeter, ECG signals were continuously acquired for 24 h in conscious rats. Terminal experiments including measurement of inducibility of ventricular arrhythmia, measurement of vagal control of ventricular function, hemodynamic and morphological measurements, and patch-clamp recording for N-type Ca^2+^ currents were performed at 3, 8, and 14 weeks after left coronary ligation (CHF groups) or sham surgery (Sham groups), respectively.

Study design, timeline, and interventions are shown in Figure 1. In the present study, 45 male Sprague-Dawley rats (6–7 weeks of age, 180–200 g) were randomly assigned to one of two groups: sham (*n* = 15) and CHF (*n* = 30). All rats were housed two per cage under controlled temperature and humidity, a 12:12-h dark-light cycle, and provided water and rat chow *ad libitum*. CHF rats were anesthetized with 2% isoflurane (Butler Schein Animal Health, Dublin, OH, USA) for surgical ligation of the left coronary artery, and sham rats underwent the same surgery without coronary ligation, as described previously (Zhang et al., 2014, 2015). In the terminal experiment, a Millar pressure transducer was used to determine LV end-diastolic pressure (LVEDP) and systolic pressure (LVSP). A colorimetric technique was used to measure infarct size (see detail below). Rats with both LVEDP >15 mmHg and infarct size >30% of the left ventricle were considered as CHF. Fifteen rats in CHF groups were excluded from the study, wherein 7 rats died within 1 week after coronary ligation, 5 rats died within 12–14 weeks after coronary ligation, and 3 rats were not considered as CHF due to insufficient LVEDP and/or infarct size. The cause of animal deaths is not clear in the present study because we did not perform measurement of the ECG and cardiac function in those dead rats.

### Implantation of the ECG telemeter and labeling of AVG neurons

Implantation of the ECG telemeter (Millar Instruments, Houston, TX, USA) was performed as described previously (Opitz et al., 1995; Baltogiannis et al., 2005; Shiba et al., 2012). As shown in Figure 1, on the day following 2, 7, or 13 weeks of coronary ligation (CHF groups) or the same surgery without coronary ligation (sham groups), rats were anesthetized with 2% isoflurane (Butler Schein Animal Health, Dublin, OH, USA), and skin was shaved and sterilized. After laparotomy was performed at the Linea Alba (abdomen), an ECG transmitter was placed into the abdominal cavity and secured to the abdominal wall at the best position for battery recharging and signal communication. In accordance with the Millar User Manual for ECG recording, bipolar electrodes were tunneled subcutaneously. The negative electrode was secured in the upper sternal midline and the positive electrode was attached to the underlying tissue near the left side of the xiphoid process. To reduce electrical noise during ECG recording, electrodes were kept together and run alongside each other as far as practical. All incisions were sutured in two layers. ECG recording was performed after 1 week recovery.

Although the ventricular myocardium only receives projection of nerve terminals from the AVG (Pardini et al., 1987), it is possible that the AVG also innervates other parts of the heart. To explore the relationship between parasympathetic activity and ventricular arrhythmia, we used a transported flourescent dye (red color DiI) to retrograde-label AVG neurons projecting to the ventricular myocardium. After implantation of the ECG telemeter was completed, a left thoracotomy was performed in the fourth intercostal space. Eight injections (2 μl DiI for each injection) were made subepicardially into the left ventricle, using a fine-tipped glass micropipette connected to a microinjector (Nanoliter 2000, WPI, Sarasota, FL, USA; Pardini et al., 1987). The surgical incision was closed and terminal experiments were performed at least 1 week after surgery, to allow the dye to diffuse to the neurons.

### ECG recording in conscious rats

One week after implantation of the ECG telemeter, rats were placed on a SmartPad receiver (Millar Instruments, Houston, TX, USA). For quantification of ventricular arrhythmic events, 24-h continuous ECG signals were acquired in unrestrained, conscious rats. Real-time ECG signals were digitalized and analyzed by PowerLab 8/30 Data Acquisition System with LabChart 7 software and ECG analysis module (ADInstruments, Colorado Springs, CO, USA). The number of premature ventricular contractions (PVCs) and cumulative duration of VT/VFs were counted manually during 24-h continuous ECG recording. VT was defined as PVCs lasting ≥4 beats. VF was defined as rapid, irregular QRS complexes. QT and QTc intervals as well as dispersions (QT_d_ and QTc_d_) were calculated from ECG recordings using Labchart software (Costa et al., 2008). QTc interval was calculated by Bazett's formula (QT$\sqrt{RR}$, where RR is RR interval; Costa et al., 2008). As an index of the spatial dispersion of the ventricular repolarization, QT_d_ and QTc_d_ were calculated by equations: QT_d_ = QT_max_ − QT_min_ and QTc_d_ = QTc_max_ − QTc_min_, where QT_max_ and QTc_max_ are the maximum QT interval and the maximum QTc interval; QT_min_ and QTc_min_ are the minimum QT interval and the minimum QTc interval (Costa et al., 2008). T-peak to T-end interval (Tpe), another marker of transmural dispersion of the ventricular repolarization, was calculated to serve as an ECG marker of ventricular arrhythmia (Yan et al., 2003; Antzelevitch, 2007; Yagishita et al., 2015).

### Measurement of inducibility of ventricular tachyarrhythmia in anesthetized rats

One day after radiotelemetry ECG recording, rats were anesthetized (800 mg/kg urethane combined with 40 mg/kg α-chloralose, i.p.), and the trachea was cannulated to facilitate mechanical respiration. Animal body temperature was maintained at 37°C with an animal temperature controller (ATC 1000; World Precision Instruments, Sarasota, FL, USA). Surface lead-II ECG was recorded using subcutaneous electrodes connected to a biological amplifier (AD Instruments, Colorado Springs, CO, USA). A left thoracotomy was then performed in the fourth intercostal space. After the heart was visualized, the pericardium was carefully removed. A bipolar platinum stimulating electrode was placed on the right ventricular outflow tract for electrical stimulation (Kang et al., 2009). Programmed electrical stimulations (PES) were performed by a programmed electrical stimulator (Digital Pulse Generator 1831; WPI, USA) and an isolator (A320R Isostim Stimulator; WPI, USA). The pulse current output was set to a twice capture threshold and a 2-ms pulse width. To determine the ventricular effective refractory period (VERP), a train of eight stimuli (8 × S1) at a 120 ms cycle length was applied, followed by an extra stimulus (S2). Starting at 90 ms, the S1–S2 interval was reduced in steps of 2 ms until the VERP was identified (Gui et al., 2013). Based on the VERP, a programmed stimulation protocol combined by single (S2), double (S3) or triple extra- stimulus (S4) after a train of eight stimulus (8 × S1) was designed to induce ventricular tachyarrhythmia as described previously (Kang et al., 2009; Shiba et al., 2012; Hong et al., 2014). The end point of ventricular pacing was induction of ventricular tachyarrhythmia. Ventricular tachyarrhythmia was considered as non-inducible when PES induced either no ventricular premature beats or self-terminated ventricular premature beats <6. Ventricular tachyarrhythmia was considered as nonsustained when it lasted ≤15 beats and sustained when it lasted >15 beats before spontaneously terminating (Belichard et al., 1994; Nguyen et al., 1998).

Inducibility of ventricular tachyarrhythmia was quantified by a quotient of ventricular arrhythmic score as described previously (Nguyen et al., 1998; Kang et al., 2009): 0, non-inducible preparations; 1, nonsustained tachyarrhythmias induced with 3 extra-stimuli; 2, sustained tachyarrhythmias induced with 3 extra-stimuli; 3, non-sustained tachyarrhythmias induced with 2 extra-stimuli; 4, sustained tachyarrhythmias induced with 2 extra-stimuli; 5, non-sustained tachyarrhythmias induced with 1 extra-stimulus; 6, sustained tachyarrhythmias induced with 1 extra-stimulus; 7, tachyarrhythmias induced during a train of 8 stimuli (8 × S1) at a basic cycle length of 120 ms; 8, the heart stopped before the PES.

### Measurement of hemodynamics and vagal control of ventricular function

After inducibility of ventricular arrhythmia was detected, the left femoral artery was cannulated with a polyethylene-50 catheter for measurement of blood pressure. A Millar pressure transducer (SPR 524; size, 3.5-Fr; Millar Instruments, Houston, TX, USA) was slowly inserted into the right carotid artery and carefully advanced to the left ventricle for measurement of left ventricular systolic pressure (LVSP), the maximum rate of left ventricular pressure rise (LV dP/dt_max_), and left ventricular end-diastolic pressure (LVEDP). Hemodynamic data were recorded by PowerLab 8/30 Data Acquisition System with LabChart 7 software (ADInstruments, Colorado Springs, CO, USA). Then, bilateral cervical vagal nerves, sympathetic nerves, and aortic depressor nerves (an afferent branch of the vagal nerve innervating the aortic arch and thoracic aorta) were isolated and transected to avoid influence of the arterial baroreflex. Because we found that response of LVSP to left vagal efferent nerve stimulation was markedly stronger than that to right vagal efferent nerve stimulation, the peripheral end of the left vagal nerve was placed on a bipolar stimulating electrode for vagal efferent nerve stimulation. Left vagal efferent nerve stimulation was applied by a Grass S9 stimulator (Grass instruments, Quincy, MA, USA) with 10 s of constant-frequency stimulation (0.1 ms pulse duration and intensity of 7.5 V at 1–100 Hz). As the index of vagal control of ventricular function, changes of LVSP and LV dP/dt_max_ in response to different frequencies of left vagal efferent nerve stimulation were recorded by PowerLab 8/30 data acquisition system with LabChart 7 software (ADInstruments, Colorado Springs, CO, USA).

### Morphological measurements

After *in-vivo* experiments were performed, the rat heart was removed to measure infarct size. A digital image of the left ventricle was captured by a digital camera (Canon, Japan). Infarct size was determined using a colorimetric technique coupled to a computerized planimetric analysis (Adobe Photoshop CS5 Extended). The percentage of infarct area to whole left ventricle was quantified using Adobe Photoshop CS5 Extended (Adobe Systems Incorporated, CA).

### Isolation of AVG neurons and whole cell patch-clamp recording for Ca^2+^ currents

After *in-vivo* experiments were performed, the AVG, located in a white color epicardial adipose pad at the junction of inferior pulmonary veins and left atrium, was exposed. AVG neurons were isolated by two-step enzymatic digestion protocol, as described previously (Liu et al., 2012; Tu et al., 2014). Briefly, the AVG was minced with microscissors and incubated with a modified Tyrode's solution containing 0.1% collagenase and 0.1% trypsin for 30 min at 37°C. The tissue was then transferred to a modified Tyrode's solution containing 0.2% collagenase and 0.5% bovine serum albumin for 30 min of incubation at 37°C. The isolated neurons were cultured at 37°C in a humidified atmosphere of 95% air-5% CO_2_ for 4–8 h before patch-clamp experiments.

Voltage-gated Ca^2+^ currents were recorded only in DiI-labeled AVG neurons (ventricular vagal neurons) by the whole cell patch-clamp technique using an Axopatch 200B patch-clamp amplifier (Axon Instruments; Tu et al., 2014). Resistance of the patch pipette was 4–6 MΩ when filled with the following solution (in mM): 120 CsCl, 1 CaCl2, 40 HEPES, 11 EGTA, 4 MgATP, 0.3 Tris-GTP, 14 creatine phosphate, and 0.1 leupeptin (pH 7.3; 305 mosM). The extracellular solution consisted of (in mM): 140 TEA-Cl, 5 BaCl2, 1 MgCl2, 10 HEPES, 0.001 TTX, 2 4-AP, and 10 glucose (pH 7.4; 310 mosM). Series resistance of 5–13 MΩ was electronically compensated at 30–80%. Junction potential was calculated to be +7.9 mV using the P-clamp 10.2 program (Axon Instruments), and all values of membrane potential given throughout were corrected using this value. Current traces were sampled at 10 kHz and filtered at 5 kHz. The holding potential was −80 mV and current-voltage (I−V) relationships were elicited by 5-mV step increments to potentials between −60 and 60 mV for 500 ms. Peak currents were measured for each test potential, and current density was calculated by dividing peak current by cell membrane capacitance. The P-clamp 10.2 program (Axon Instruments) was used for data acquisition and analysis. All experiments were performed at room temperature (22–24°C). In patch clamp experiments, ω-conotoxin GVIA (Alomone Labs) was used to block N-type Ca^2+^ channels. Based on the previous study, the concentration of ω-conotoxin GVIA (1 μM) used in the present study is a saturating concentration for inhibiting N-type Ca^2+^ channels (Jeong and Wurster, 1997; Tu et al., 2014).

### Statistical analysis

All data are presented as means ± SEM. SigmaPlot 12 was used for data analysis. Statistical significance was determined by a two-way ANOVA with a *post-hoc* Bonferroni test for multi group comparison, a Fisher exact test for the incidence of ventricular arrhythmias, and a spearman rank correlation analysis for the correlation of ventricular arrhythmogenesis with N-type Ca^2+^ currents. Normal distribution of data was confirmed with the Kolmogorov-Smirov test and equal variance with Levene's test. Statistical significance was accepted when *p* < 0.05.

## Results

### Hemodynamic and morphological characteristics in sham and CHF rats

The hemodynamic and morphological characteristics from sham and CHF rats are summarized in Table 1. There was no significant difference in mean arterial pressure (MAP) between age-matched sham and CHF rats (Table 1). As the index of left ventricular contractility, LVSP and LV dP/dt_max_ were gradually decreased from the early stage (3 weeks after MI, 3-week CHF) to the late stage of CHF rats (14 weeks after MI, 14-week CHF), compared with age matched sham rats (*p* < 0.05 vs. age-matched sham rats). LVEDP was also significantly elevated in 3, 8, and 14-week CHF rats. In CHF rats, a gross examination displayed a dense scar in the anterior ventricular wall and myocardial infarct size about 40% of the left ventricular area, regardless whether 3, 8, or 14-week CHF rats were observed (*p* < 0.05 vs. age-matched sham rats).

**Table 1:** **Hemodynamic and morphological characteristics in anesthetized sham and CHF rats**.

	**Sham**			**CHF**		
	**3-weeks**	**8-weeks**	**14-weeks**	**3-weeks**	**8-weeks**	**14-weeks**
MAP (mmHg)	101.4 ± 4.0	103.8 ± 4.6	102.8 ± 4.4	103.2 ± 4.4	102.4 ± 4.3	104.2 ± 4.9
Infarct size (% of LV)	0	0	0	37.4 ± 2.2^*^	39.0 ± 3.1^*^	38.2 ± 2.1^*^
LVSP (mmHg)	121.8 ± 3.9	122.6 ± 3.8	122.8 ± 3.4	115.2 ± 4.2^*^	112.2 ± 4.6^*^	101.2 ± 3.7^*^
LV dP/dt_max_ (mmHg/s)	6165 ± 278	6023 ± 274	6132 ± 241	5605 ± 244	5249 ± 228^*^	4894 ± 287^*^
LVEDP (mmHg)	2.1 ± 0.4	2.0 ± 0.4	2.3 ± 0.4	17.0 ± 0.7^*^	17.6 ± 1.1^*^	18.4 ± 1.5^*^

*CHF, chronic heart failure; MAP, mean arterial pressure; LVSP, left ventricular systolic pressure; LV dP/dt_max_, the maximum rate of left ventricular pressure rise; LVEDP, left ventricular end-diastolic pressure. Data are means ± SEM; n = 5 in each group*.

* *P < 0.05 vs. age-matched sham rats*.

### Spontaneous ventricular arrhythmias during the development of heart failure in conscious rats

**Figure 2:**
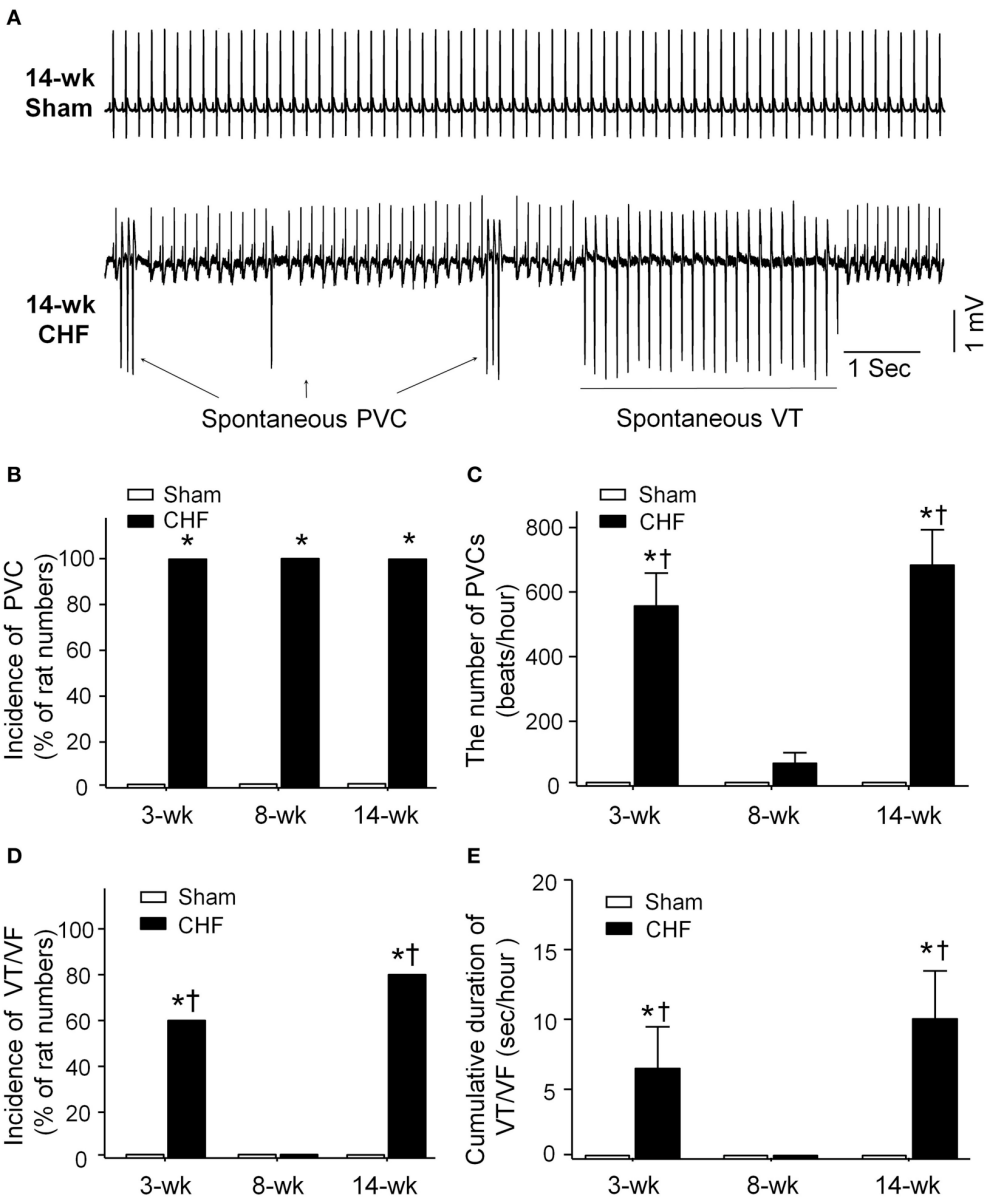
**Data for spontaneous arrhythmias obtained from 24-h continuous telemetry ECG recording in conscious sham and CHF rats. (A)** Representative ECG images showing ventricular arrhythmias including spontaneous premature ventricular contractions (PVCs) and ventricular tachycardia (VT) occurred in the late stage of CHF rat (14-week CHF). **(B,C)** Incidence and the number of PVCs in 3, 8, and 14-week sham and CHF rats. **(D,E)** Incidence and cumulative duration of VT/VF in 3, 8, and 14-week sham and CHF rats. Data are mean ± SEM; *n* = 5 rats in each group. ^*^P < 0.05 vs. age-matched sham rats; ^†^*p* < 0.05 vs. 8-week CHF rats.

Spontaneous ventricular arrhythmias during the progression of conscious CHF rats were monitored by radiotelemetry ECG recording (Figure 2). Neither PVCs nor VT/VF was observed in sham rats. Although all rats had PVCs in all stages of CHF (Figure 2B), the number of PVCs was markedly increased only in 3 and 14-week CHF rats (Figure 2C). Additionally, the incidence and cumulative duration of VT/VF calculated from 24-h continuous ECG recording were significantly raised in 3 and 14-week CHF rats, whereas there was non-occurrence of VT/VF in 8-week CHF rats (Figures 2D,E). These results indicate that malignant ventricular arrhythmias mainly occur in both early and late stages of MI-induced CHF rats.

### ECG analysis from continuous radiotelemetry ECG recording in conscious sham and CHF rats

**Figure 3:**
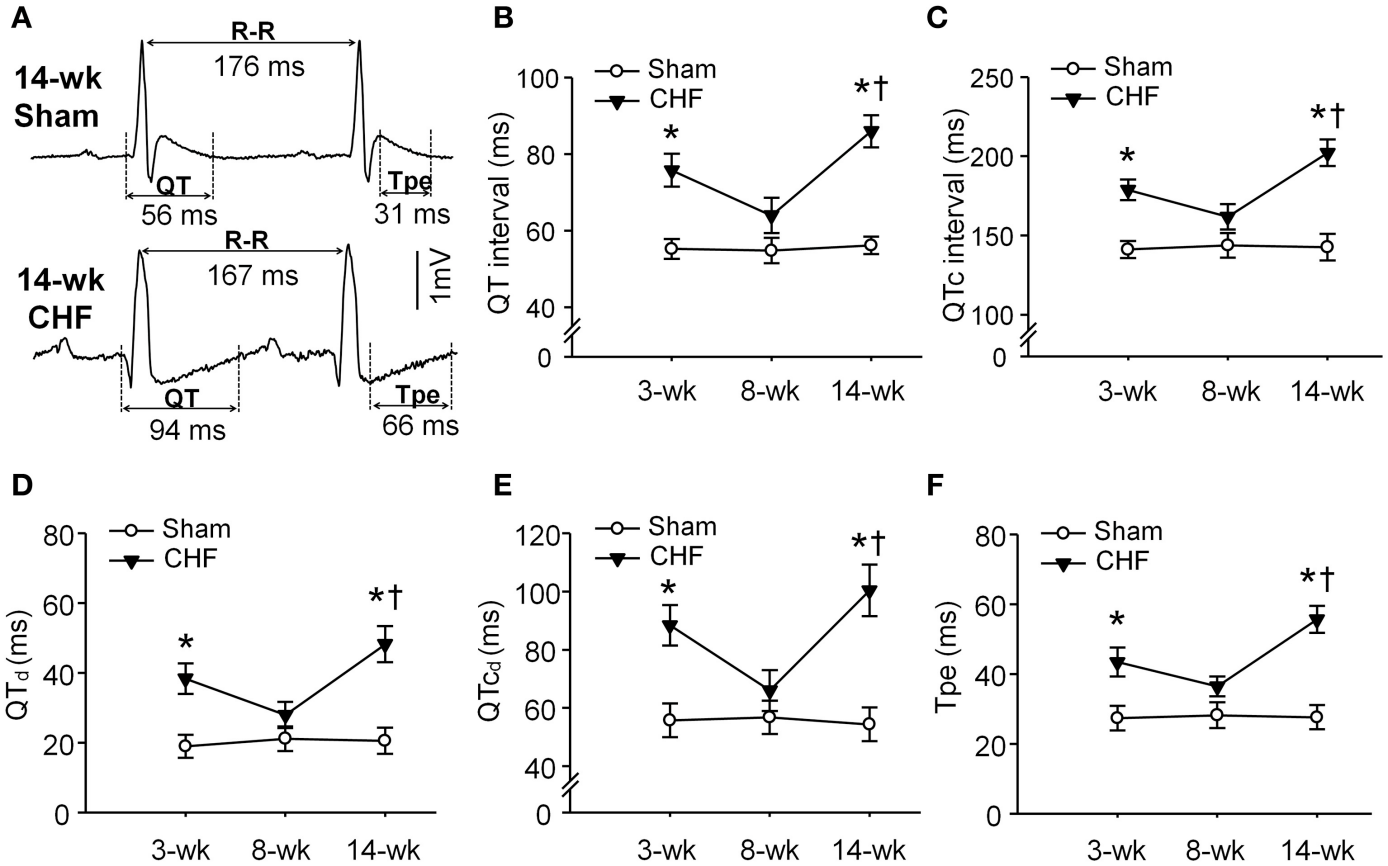
**Ventricular electrical activities calculated from 24-h continuous telemetry ECG recording in conscious sham and CHF rats. (A)** Representative lead-II ECG images illustrating prolongation of QT and T-peak to T-end interval (Tpe) in 14-week CHF rats. **(B–F)** Mean data for QT interval, corrected QT (QTc) interval, QT dispersions (QT_d_), QTc dispersions (QTc_d_), and Tpe in 3, 8, and 14-week sham and CHF rats. Data are mean ± SEM; *n* = 5 rats in each group. ^*^P < 0.05 vs. age-matched sham rats; ^†^*p* < 0.05 vs. 8-week CHF rats.

To determine whether ventricular arrhythmogenesis in both early and late stages of CHF rats was associated with heterogeneity of ventricular electrical activity, ECG markers of ventricular arrhythmogenesis, including QT, QTc, QT_d_, QTc_d_, and Tpe were evaluated in conscious sham and CHF rats. All ECG parameters (QT, QTc, QT_d_, QTc_d_, and Tpe) were significantly prolonged in 3 and 14-week CHF rats, compared to age-matched sham rats (Figure 3). However, these ECG markers of ventricular arrhythmogenesis did not show marked changes in 8-week CHF rats (*P* > 0.05 vs. age-matched sham rats).

### Susceptibility to ventricular arrhythmias in anesthetized sham and CHF rats

**Figure 4:**
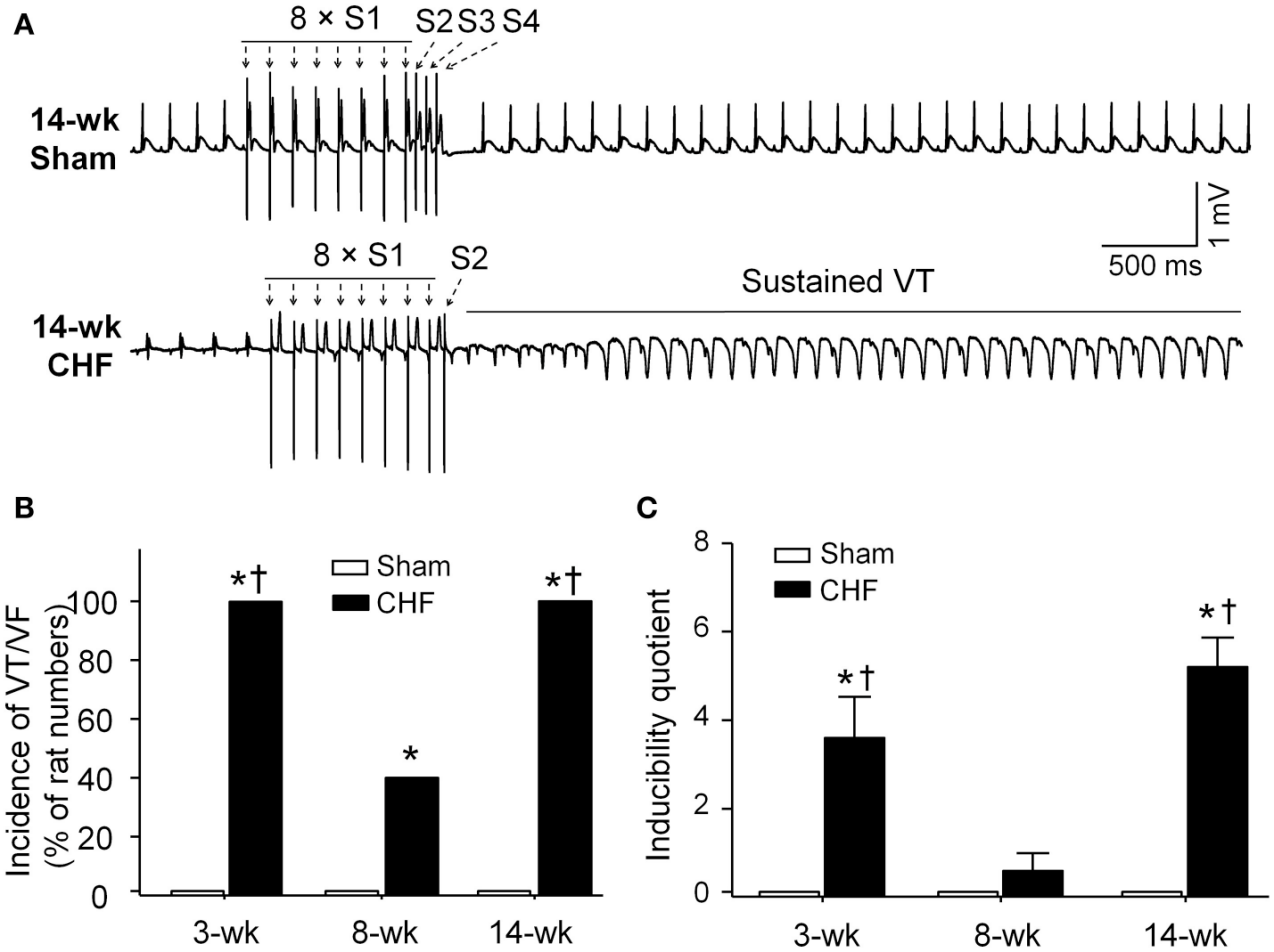
**Inducibility of ventricular tachyarrhythmia evoked by programed electrical stimulation (PES) in anesthetized sham and CHF rats. (A)** Representative ECG recordings showing nonoccurrence of PES-evoked ventricular arrhythmias in sham rat, and induction of VT evoked by PES in 14-week CHF rat. **(B,C)** Incidence and inducibility quotient of ventricular arrhythmias in 3, 8, and 14-week sham and CHF rats. Data are mean ± SEM; *n* = 5 rats in each group. ^*^P < 0.05 vs. age-matched sham rats; ^†^*p* < 0.05 vs. 8-week CHF rats.

In sham rats, ventricular arrhythmias were not induced by S_1_S_2_S_3_S_4_ programmed stimulation. Inducibility quotient of ventricular arrhythmias was zero in sham rats (Figure 4). In 3 and 14-week CHF rats, 100% (5/5) of the rats had PES-induced VT/VF, and inducibility quotient of ventricular arrhythmias was 3.6 ± 0.9 and 5.2 ± 0.7, respectively (*p* < 0.05 vs. age-matched sham rats). In 8-week CHF rats, inducibility quotient of ventricular arrhythmias was 0.6 ± 0.4 (*p* > 0.05 vs. age-matched sham rats), although PES-induced VT/VF occurred in 40% (2/5) of the rats (*p* < 0.05 vs. age-matched sham rats, Figure 4). These data further confirm that malignant ventricular arrhythmias readily occur in the early and late stages of MI-induced CHF rats.

### Changes in vagal control of the ventricle during the development of CHF

**Figure 5:**
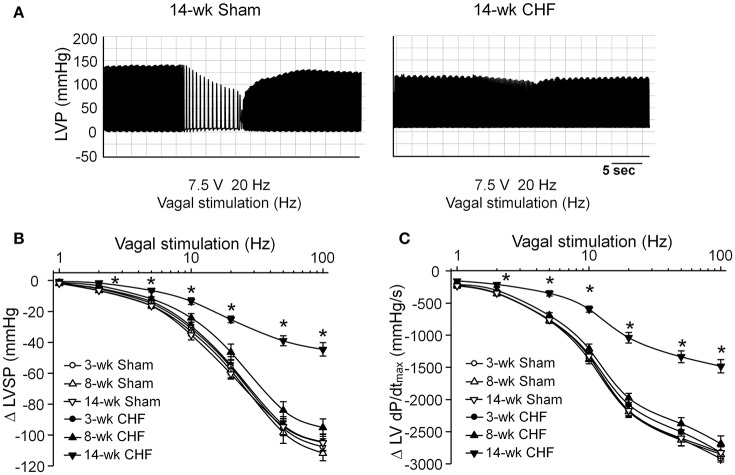
**Vagal control of ventricular function measured by changes of left ventricular systolic pressure (LVSP) in response to different frequencies of left vagal efferent nerve stimulation in anesthetized sham and CHF rats. (A)** Representative recordings demonstrating change of LVSP in response to 20 Hz of left vagal efferent nerve stimulation in 14-week sham and CHF rats. **(B,C)** Quantitative data for changes of LVSP and LV dP/dt_max_ in response to different frequencies (1–100 Hz) of left vagal efferent nerve stimulation in 3, 8, and 14-week sham and CHF rats. Data are mean ± SEM; *n* = 5 rats in each group. ^*^P < 0.05 vs. age-matched sham rats.

Activation of the vagal efferent nerve results in negative inotropic effects in the ventricle, which serves as an index for ventricular response to vagal stimulation. Changes of the LVSP and LV dP/dt_max_ in response to different frequencies of vagal efferent nerve stimulation were markedly blunted in 14-week CHF rats, compared to age-matched sham rats (*p* < 0.05, Figure 5). However, there were no significant changes in these parameters in 3 and 8-week CHF rats (*p* > 0.05 vs. age-matched sham rats, Figure 5).

### N-type Ca^2+^ currents in AVG neurons from sham and CHF rats

**Figure 6:**
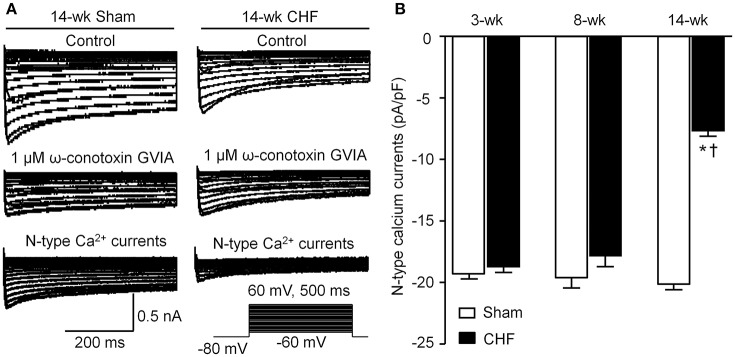
**N-type Ca^**2+**^ channel currents in DiI-labeled AVG neurons from sham and CHF rats. (A)** Original recordings of Ca^2+^ currents in DiI-labeled AVG neurons from 14-week sham and CHF rats. N-type Ca^2+^ currents were obtained by subtracting Ca^2+^ currents under treatment of ω-conotoxin GVIA from total Ca^2+^ currents. **(B)** Mean data for N-type Ca^2+^ currents in DiI-labeled AVG neurons from 3, 8, and 14-week sham and CHF rats, measured in response to a test pulse from −80 to −10 mV. Data are mean ± SEM; *n* = 25 neurons from 5 rats in each group. ^*^P < 0.05 vs. age-matched sham rats; ^†^*p* < 0.05 vs. 3 and 8-week CHF rats.

Voltage-gated Ca^2+^ currents in DiI-labeled AVG neurons (ventricular vagal neurons) were recorded by whole-cell patch-clamp technique. A specific N-type Ca^2+^ channel blocker (1 μM ω-conotoxin GVIA) was used to separate N-type Ca^2+^ currents from total Ca^2+^ currents. N-type Ca^2+^ currents were obtained by subtracting Ca^2+^ currents under treatment of ω-conotoxin GVIA from total Ca^2+^ currents (Figure 6A). There was no significant alteration in N-type Ca^2+^ currents in AVG neurons from 3 and 8-week CHF rats, compared to age-matched sham rats (*p* > 0.05, Figure 6B). Fourteen-week CHF significantly decreased N-type Ca^2+^ currents in AVG neurons (*p* < 0.05 vs. age-matched sham rats, Figure 6).

### Correlation of ventricular arrhythmogenesis with N-type Ca^2+^ currents in AVG neurons during the progression of CHF

**Figure 7:**
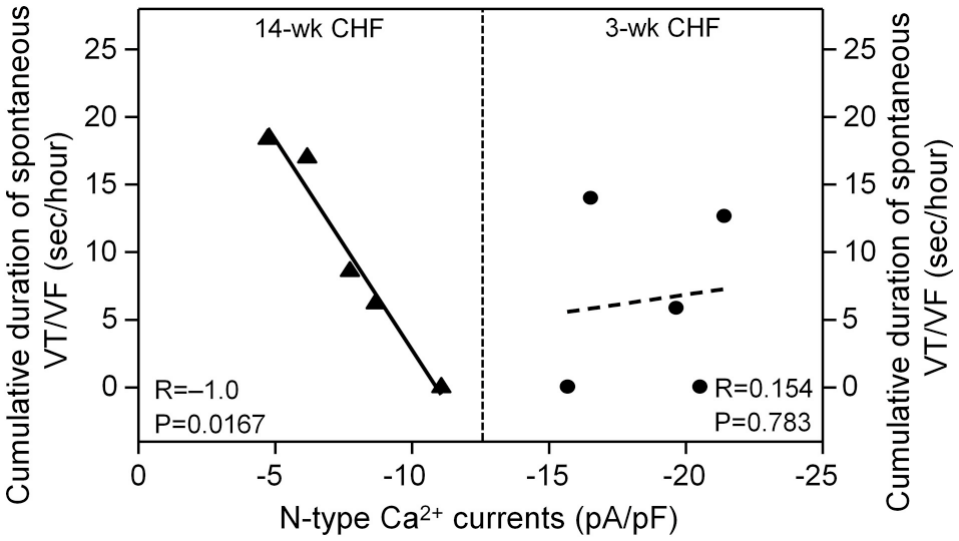
**Correlation between duration of spontaneous VT/VF and N-type Ca^**2+**^ currents in AVG neurons in 3 and 14-week CHF rats**. R is the correlation coefficient. *N* = 25 neurons from 5 rats in each group. Statistical significance was accepted when *p* < 0.05.

Since malignant ventricular arrhythmias occurred in 3 and 14-week CHF rats, correlation between duration of VT/VF and N-type Ca^2+^ currents in AVG neurons was evaluated in 3 and 14-week CHF rats. As shown in Figure 7, N-type Ca^2+^ currents in AVG neurons negatively correlated with duration of VT/VF in 14-week CHF rats (*R* = −1.0, *P* = 0.0167). However, there was no significant correlation between N-type Ca^2+^ currents in AVG neurons and duration of VT/VF in 3-week CHF rats (*R* = 0.154, *P* = 0.783).

## Discussion

Our current study demonstrates the correlation between electrical remodeling of AVG neurons and ventricular arrhythmogenesis in different stages of CHF. ECG data obtained from both continuous telemetry recording in conscious rats and PES-induced inducibility of ventricular arrhythmias in anesthetized rats showed that malignant ventricular arrhythmias readily occurred in the early stage (3-week CHF) and late stage (14-week CHF) of CHF rats. Ventricular response to vagal nerve stimulation was markedly blunted in 14-week CHF rats but not in 3 and 8-week CHF rats. Whole-cell patch-clamp data showed that N-type Ca^2+^ currents in AVG neurons were decreased in 14-week CHF rats. More importantly, the data from correlation analysis confirmed that electrical remodeling of AVG neurons negatively correlated with the occurrence of malignant ventricular arrhythmias in 14-week CHF rats but not in 3-week CHF rats. The above results suggest that decrease in N-type Ca^2+^ currents in AVG neurons might be associated with ventricular arrhythmias at late stage CHF.

Malignant ventricular arrhythmias remain a leading cause of SCD in patients with CHF (Tomaselli and Zipes, 2004; Kolettis et al., 2015). In the present study, malignant ventricular arrhythmias mainly occurred in early and late stage CHF, whereas they did not occur in intermediate stage CHF (Figure 2). A hallmark of CHF is autonomic nervous dysfunction, including cardiac sympathetic overactivation and withdrawal of cardiac parasympathetic activity (Saul et al., 1988; Porter et al., 1990; Floras, 1993, 2009; Triposkiadis et al., 2009; Schwartz and De Ferrari, 2011). In addition to structural and electrophysiological remodeling of the left ventricle, growing evidence has demonstrated that sympathetic overactivation is thought to be an important ventricular arrhythmogenic mechanism during the progression of CHF, especially in the early stage of CHF (Meredith et al., 1991; Du et al., 1999; Aronson and Burger, 2003; Watson et al., 2006; Zipes and Rubart, 2006; Kolettis et al., 2015; Ajijola et al., 2017). However, we cannot ignore the role of attenuated cardiac parasympathetic activity in ventricular arrhythmogenesis in CHF. Some previous studies have shown that impairment of cardiac parasympathetic activation is associated with ventricular arrhythmia-related mortality in the CHF state (Corr and Gillis, 1974; Nolan et al., 1998; Frenneaux, 2004; Li et al., 2004; Zipes and Rubart, 2006; Cygankiewicz et al., 2008; Hauptman et al., 2012; Shen and Zipes, 2014). Our present study found that ventricular response to vagal nerve stimulation was attenuated in the late but not the early stage of CHF rats, although malignant ventricular arrhythmias occurred in both early and late stage CHF (Figures 2, 5). These results indicate that attenuated ventricular vagal activation might be associated with the occurrence of malignant ventricular arrhythmias in late stage CHF.

Cardiac vagal preganglionic fibers originate within the central nervous system at the level of the brainstem (nucleus ambiguous and nucleus tractus solitaries; Olshansky et al., 2008; Thomas, 2011). These vagal preganglionic efferent fibers extend to the intracardiac ganglia, located in cardiac fat pads, to form synapses with vagal postganglionic neurons (Olshansky et al., 2008; Thomas, 2011). Cardiac vagal postganglionic neurons located in the intracardiac ganglia provide local neural coordination independent of higher brain centers (Verrier and Antzelevitch, 2004; Cuevas, 2014). Our previous study demonstrated that a decrease in N-type Ca^2+^ currents in intracardiac ganglionic neurons reduced the neuronal excitability of intracardiac ganglionic neurons and cardiac parasympathetic abnormality in late stage CHF (Tu et al., 2014). Intracardiac ganglia are divided into the AVG and sinoatrial ganglion, and it is known that the ventricular myocardium only receives projection of nerve terminals from the AVG (Pardini et al., 1987). In the present study, we found that N-type Ca^2+^ currents in AVG neurons negatively correlated with the occurrence of ventricular arrhythmias in late stage CHF (Figure 7). Although it is unclear how electrical remodeling of AVG neurons links to ventricular arrhythmogenesis in late stage CHF, many studies (Brack et al., 2013; Aiba et al., 2015; He et al., 2016) provide strong evidence in support of the pivotal anti-arrhythmic effect of parasympathetic activation. Vagal nerve stimulation inhibits ventricular arrhythmias through direct or indirect activation of muscarinic acetylcholine receptors (Brack et al., 2013; He et al., 2016). The parasympathetic activation induced by vagal nerve stimulation or treatment with exogenous acetylcholine suppresses the occurrence of ventricular arrhythmias and mitigates SCD in CHF (Sabbah, 2011; Aiba et al., 2015). Corr et al. reported that bilateral vagotomy or treatment with atropine (a specific muscarinic acetylcholine receptor antagonist) increases MI-induced SCD (Corr and Gillis, 1974). From the results of the present study, we considered that electrical remodeling such as reduced N-type Ca^2+^ currents in AVG neurons might attenuate ventricular vagal activation and subsequently trigger ventricular arrhythmias in late stage CHF.

In the present study, first, QT and QTc intervals (the indices for total duration of the ventricular electrical activity) were markedly prolonged in the early and late stages of CHF rats (Figures 3B,C). Second, QT_d_ and QTc_d_ (the markers of spatial heterogeneity of ventricular repolarization) were increased in the early and late stages of CHF rats (Figures 3D,E). Third, a marker of transmural dispersion of ventricular repolarization, the Tpe, was prolonged in the early and late stage of CHF rats. (Figure 3F). Changes in these electrical activities represent the heterogeneity and dispersion of ventricular repolarization, and lead to malignant ventricular arrhythmias in CHF (Yan et al., 2003; Antzelevitch, 2007; Long et al., 2015; Yagishita et al., 2015; Xue et al., 2016). The action of acetylcholine binding with muscarinic acetylcholine receptors on ventricular ion channels is associated with alterations of these ventricular electrical activities (Rosenshtraukh et al., 1994; Zuberi et al., 2010; Machhada et al., 2015). The present study confirmed that electrical remodeling of AVG neurons negatively correlated with the occurrence of malignant ventricular arrhythmia in the late stage of CHF rats (Figure 7). Therefore, ventricular vagal dysfunction induced by electrical remodeling of AVG neurons may be related to changes in ventricular electrical activities and resultant malignant ventricular arrhythmias in late stage CHF.

## Limitation of the study

There are two limitations in the present study. First, although our current study demonstrates the negative correlation of ventricular arrhythmogenesis with neuronal remodeling of AVG neurons in the late stage of CHF, we could not clarify whether the occurrence of ventricular arrhythmias in the late stage of CHF is totally or partially due to neuronal remodeling of AVG neurons. Mechanical/chemical AVG destruction in a control group of sham operation possibly destroys sympathetic efferent nerve fibers passing through the AVG, which limits us from performing this measurement. An alternative way is to decrease N-type Ca^2+^ channels in sham AVG neurons using *in-vivo* lentiviral transfection of N-type Ca^2+^ channel shRNA into sham AVG neurons. We will address this issue in our future study. Second, we didn't perform echocardiography measurement in the present study because the data from echocardiography were reported in many previous studies from other investigators and ourselves (Tu et al., 2010; Del et al., 2013; Wang et al., 2014; Becker et al., 2016; Collister et al., 2016). There is no significant difference in left ventricular ejection fraction at 2–6 weeks (Wang et al., 2014; Becker et al., 2016; Collister et al., 2016), 6–8 weeks (Tu et al., 2010), and 16 weeks after left coronary ligation (Del et al., 2013). In the present study, we used elevation in LVEDP to evaluate ventricular function in different stages of CHF. Like echocardiographic data, LVEDP was elevated almost to the same level in 3, 8, and 14-week CHF rats (Table 1).

## Conclusion

Our present study demonstrates that malignant ventricular arrhythmias mainly occur in the early and late stages of MI-induced CHF. Electrical remodeling in AVG neurons might be associated with ventricular vagal dysfunction, alterations of ventricular electrical activities, and malignant ventricular arrhythmias in the late stage of CHF.

## Author contributions

DZ and YL conceived and designed the experiments. DZ, HT, CW, LC, and YL performed the experiments and analyzed the data. YL, RM, and MW contributed reagents/materials/analysis tools. DZ, RM, MW, and YL wrote the paper.

## Funding

This work was supported by American Heart Association (AHA) Grant-in-Aid (15GRANT24970002) to YL.

### Conflict of interest statement

The authors declare that the research was conducted in the absence of any commercial or financial relationships that could be construed as a potential conflict of interest.
